# Evaluation and Performance of a Positive Airway Pressure Device (CPAP-AirFlife™): A Randomized Crossover Non-Inferiority Clinical Study in Normal Subjects

**DOI:** 10.3390/medicina59081372

**Published:** 2023-07-27

**Authors:** Héctor A. Tinoco, Luis Perdomo-Hurtado, Joismer A. Henao-Cruz, José F. Escobar-Serna, Oscar Jaramillo-Robledo, Oscar D. Aguirre-Ospina, Mateo Hurtado-Hernández, Juliana Lopez-Guzman

**Affiliations:** 1 Experimental and Computational Mechanics Laboratory, Universidad Autónoma de Manizales, Antigua Estación del Ferrocarril, Edificio Fundadores, Manizales-Caldas 170001, Colombia; 2 S.E.S. Hospital Universitario de Caldas, Manizales-Caldas 170004, Colombia

**Keywords:** non-invasive ventilation (NIV), CPAP, positive airway pressure, COVID-19, mechanical ventilation

## Abstract

*Background and Objectives*: During the COVID-19, the demand for non-invasive ventilatory support equipment significantly increased. In response, a novel non-invasive ventilatory support model called CPAP-AirFlife™ was developed utilizing existing technologies. This model offers technological advantages, including an aerosol-controlled helmet suitable for high-risk environments such as ambulances. Additionally, it is cost-effective and does not require medical air, making it accessible for implementation in low-level hospitals, particularly in rural areas. This study aimed to assess the efficacy of CPAP-AirFlife™ by conducting a non-inferiority comparison with conventional ventilation equipment used in the Intensive Care Unit. *Materials and Methods*: A clinical study was conducted on normal subjects in a randomized and sequential manner. Parameters such as hemoglobin oxygen saturation by pulse oximetry, exhaled PCO_2_ levels, vital signs, and individual tolerance were compared between the CPAP-AirFlife™ and conventional equipment. The study population was described in terms of demographic characteristics and included in the analysis. *Results*: It was shown that the CPAP-AirFlife™ was not inferior to conventional equipment in terms of efficacy or tolerability. Hemoglobin oxygen saturation levels, exhaled PCO_2_ levels, vital signs, and individual tolerance did not significantly differ between the two models. *Conclusions*: The findings suggest that CPAP-AirFlife™ is a practical and cost-effective alternative for non-invasive ventilatory support. Its technological advantages, including the aerosol-controlled helmet, make it suitable for high-risk environments. The device’s accessibility and affordability make it a promising solution for implementation in low-level hospitals, particularly in rural areas. This study supports using CPAP-AirFlife™ as a practical option for non-invasive ventilatory support, providing a valuable contribution to respiratory care during the COVID-19 pandemic and beyond.

## 1. Introduction

Non-invasive ventilation (NIV) is essential for acute respiratory failure (ARF) and benefits chronic obstructive pulmonary disease (COPD), cardiogenic pulmonary edema, trauma, postoperative care, and pneumonia, reducing intubation rates, mortality, and infectious complications [1]. Moreover, NIV has been used as adjunctive therapy in conditions such as trauma, postoperative care, continuing care, and mild to moderate pneumonia cases, showing reduced intubation rates, mortality, and infectious complications [2,3]. Additionally, NIV benefits patients with chronic respiratory failure, post-tuberculosis conditions, neuromuscular diseases, and especially COPD without contraindications [3,4]. It reduces the work of breathing and alleviates muscle fatigue by aiding in alveolar unit closure during respiration [4,5]. COPD patients receiving NIV as first-line treatment have lower mortality rates than those initially undergoing endotracheal intubation (ETI) (23%, 95% CI 18–30% vs. 39%, 95% CI 33–44%). Improved pulmonary mechanics, as reflected by arterial blood gas monitoring and other parameters, may decrease intubation rates and pneumonia progression, potentially affecting ICU length of stay [6].

NIV’s impact has been studied in different indications, including immunosuppressed patients with ARF [7,8]. It has been explored for facilitating endotracheal tube removal after invasive mechanical ventilation, aiming to prevent reintubation post-extubation [7,9,10]. Current data supports NIV as an effective measure to avoid reintubation, with increasing utilization in ICU and non-ICU settings [11,12]. Patient stratification for ICU care is crucial [13]. Positive outcomes have been reported in COPD patients in pulmonology wards as well as in emergency room and intermediate care settings [11]. NIV has shown effectiveness in various lung diseases, particularly those associated with complications from invasive mechanical ventilation [12,14,15]. In late 2019, an outbreak of pneumonia of unknown cause was reported in Wuhan, China. Epithelial cells from infected patients were inoculated with respiratory samples, leading to the identification of the SARS-CoV-2 virus (COVID-19) [16]. This resulted in a significant number of patients experiencing severe acute respiratory syndrome or acute hypoxemic respiratory failure (AHRF), including mild, moderate, or severe acute respiratory distress syndrome (ARDS) [16]. The global impact of COVID-19 prompted the World Health Organization (WHO) to declare a pandemic on 12 March 2020 [17]. Various respiratory support technologies were employed to address this critical situation, such as a low-flow nasal cannula, high-flow devices, and invasive mechanical ventilation with an endotracheal tube [18,19]. One of the techniques employed for non-invasive respiratory support in addressing COVID-19 was the application of Continuous Positive Airway Pressure (CPAP), which proved beneficial in alleviating the strain on intensive care unit (ICU) beds and reducing the need for intubation [20,21]. Specific technologies were developed to minimize the dispersion of airborne particulate pollutants compared to invasive devices [22]. CPAP offers the advantage of combining a higher fraction of inspiratory oxygen with increased airway pressure, making it compatible with various sources of medical oxygen. In addition, it can be used during the weaning process of patients on invasive mechanical ventilation [22,23].

Physiologically, CPAP devices help to maintain airway patency and prevent upper airway collapse. They provide additional benefits, including increased end-expiratory lung volume, enhanced oxygen stores, and improved upper airway patency and cardiac afterload through increased tracheal traction [24]. Moreover, extensive research supports using CPAP in patients with cardiac conditions, acute pulmonary edema, secondary sleep apnea, and other related conditions [25]. CPAP is recommended in the early stages of hypoxemic respiratory failure, such as the presentation observed in the L (low) phenotype of COVID-19 pneumonia [18,26,27]. It has proven to be a valuable tool in managing respiratory distress during the COVID-19 pandemic and offers a non-invasive approach to support patients with various respiratory conditions [28].

The lack of ventilation equipment and technologies posed significant challenges for healthcare workers and patients affected by COVID-19. Consequently, the development of CPAP devices saw a significant increase due to access to additive manufacturing technologies such as 3D printing, desktop CNC, and electrical circuit printers. These advanced manufacturing methods were crucial in providing early solutions to the challenges during the COVID-19 pandemic [29,30]. Several noteworthy technologies were developed in response to this demand. For instance, a low-cost CPAP device was designed that maintains constant air pressure to support patient breathing [31]. This device utilizes a cost-effective electromechanical system consisting of an air pump and a control unit. Preliminary evaluations of this device demonstrated functionality and performance characteristics similar those of other standard CPAP devices. In collaboration with Coeo Labs (Coeo Laboratory division of InnAccel Technologies Pvt Ltd., Bangalore, India), [32] developed “Saans”, a versatile, user-friendly, and cost-effective device. “Saans” focuses on developing a conventional method of air-oxygen mixing using CPAP technology. It operates in multiple modes, drawing ambient air and medical oxygen to deliver the required oxygen concentration. Among the most significant advancements was the UCL-Ventura CPAP designed by the University College of London Hospital (UCLH) and manufactured in collaboration with Mercedes-AMG High Performance Powertrains This partnership resulted in the development of a notable CPAP device that addressed critical needs during the pandemic. (HPP, Brixworth, Northants, UK) [33]. It was demonstrated that non-invasive ventilatory support devices such as CPAP can significantly reduce the need for invasive mechanical ventilation in COVID-19 patients [34]. Additionally, the use of CPAP helmets was investigated in COVID-19 patients, with improved oxygenation and a reduced requirement for invasive mechanical ventilation observed by Bellani [35]. The UCL-Ventura CPAP is based on the WhisperFlow flow generator [36] originally invented by Medic-Aid (Pagham, Sussex, UK) in 1992. During the COVID-19 pandemic, the device was utilized for patients across various countries, initially in the UK. The release of detailed designs and manufacturing procedures enabled its replication and modification on a global scale. The CPAP-AirFlife™ model proposed in this study, is one such modification of the UCL-Ventura device, featuring new size and functionality optimizations. 

Notably, under COVID-19 conditions the CPAP-AirFlife™ fulfills the requirements for continuous airway pressure delivery via mask and incorporates necessary biosafety measures to control potential aerosol generation. This device has been classified as an emergency device and should only be used under the supervision of trained healthcare professionals [37]. The CPAP-AirFlife™ system has received approval from the INVIMA (National Institute of Drugs and Food Surveillance), enabling its production and marketing under health registration No. 2021058033, issued on 23 December 2021.

The CPAP-AirFlife™ non-invasive Ventilatory Support System has emerged as an effective technological and clinical alternative in response to the COVID-19 pandemic. This device can potentially prevent disease progression to severe respiratory failure, improve blood oxygen levels, and mitigate alveolar collapse in patients with mild to moderate respiratory distress, as indicated by previous studies. The CPAP-AirFlife™ system offers additional advantages, including affordability, portability, and ease of use, enabling its application in ambulances and across various levels of care and hospital services, including in rural areas. It is able to facilitate ventilation in patients with a variety of respiratory conditions. 

To assess its performance, a series of experimental tests were conducted to characterize the ventilation circuit parameters. A non-inferiority test was carried out on healthy patients at the S.E.S Hospital Universitario de Caldas to evaluate whether the technology meets the standards of mechanical ventilation equipment used in the ICU. 

## 2. Materials and Methods

### 2.1. Description of the CPAP-AirFlife™ Device

**Figure 1:**
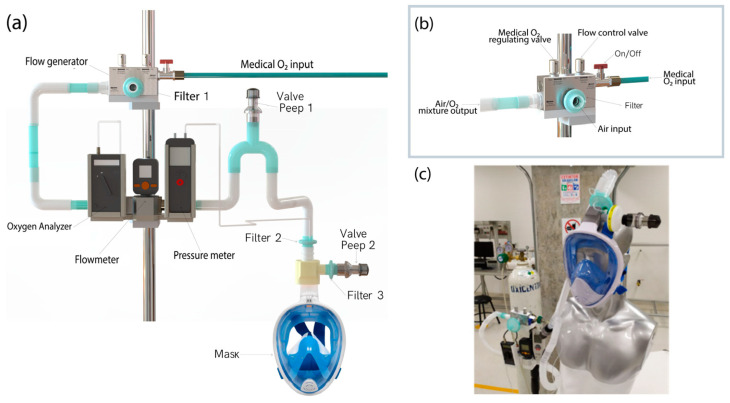
(**a**) CPAP-AirFlife™ ventilatory system. (**b**) Flow generator. (**c**) Application of the CPAP-AirFlife™.

Figure 1a illustrates the CPAP-AirFlife™ non-invasive ventilation system designed to establish positive airway pressure (CPAP) and maintain a consistent positive end-expiratory pressure (PEEP) throughout the respiratory cycle. The CPAP-AirFlife™ system comprises several components: a flow generator based on the UCL-Ventura generator developed by the University College of London Hospital (UCLH) in collaboration with Mercedes-AMG High Performance Powertrains [33], a one-way ventilatory circuit, three antibacterial filters, two PEEP valves, a ventilation mask connector, a two-way ventilatory circuit, an on/off valve, a flow generator holder, a bank of sensors (including a flowmeter, digital manometer, and oxygen analyzer), and an adult ventilation mask. The CPAP-AirFlife™ system receives regulated medical oxygen from a dedicated line that enters through the flow generator. This device effectively regulates the flow of air/O_2_ mixture and controls the oxygen concentration. It is essential to note that the CPAP-AirFlife™ system requires medical oxygen as an energy source. Oxygen is directed through the flow generator, which combines with filtered atmospheric air through a venturi mechanism in the suction chamber, as depicted in Figure 1b. During this process, atmospheric air is drawn into the internal chamber at a pressure lower than atmospheric pressure to ensure a constant flow of air/O_2_ mixture free from biological and particulate matter. As with other types of flow generators analyzed in the literature, the flow rate can be up to 100 L per minute at an inlet setting of 50 psi, and the oxygen concentration can be from 40% to 100% [38,39]. The generator valves can control both parameters, allowing the setting of the desired values measurements from an oxygen sensor and a flow meter. When the controlled air–oxygen mixture is generated, it is directed to the patient circuit, which includes a 20 cmH_2_O PEEP No. 1 valve acting as a safety valve to prevent lung injury before the flow reaches the ventilation mask [40].

CPAP-AirFlife™ non-invasive ventilatory support system comprises an inspiratory and expiratory airway mask. Positioned at the end of the inspiratory line is a PEEP No. 2 valve that maintains positive airway pressure, which can be adjusted based on the patient’s condition, as shown in Figure 1c. The expiratory pressure value can range from 3–20 cmH_2_O [41]. While the PEEP No. 2 valve does not have a fixed pressure value, increasing the flow rate allows for an increase in pressure. Therefore, the pressure sensor aids in determining the appropriate basal level required for CPAP therapy, enhancing the system’s safety and effectiveness [40].

### 2.2. Functional Validation of the CPAP-AirFlife™ Flow Generator

**Figure 2:**
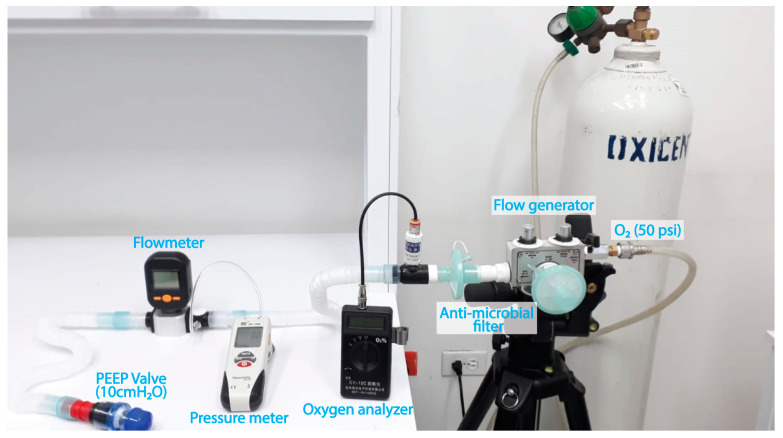
Configuration for validation of the CPAP-AirFlife™ flow generator.

This section provides a comprehensive overview of the tests conducted to validate the flow generator, a crucial component responsible for controlling flow and oxygen concentrations in the device. The testing process involved meticulously examining the flow generator valve performance, oxygen concentration, flow rates, and PEEP pressure parameters within a closed circuit with limited ventilation and a PEEP valve. A well-designed test rig was assembled to ensure accurate and reliable results, as depicted in Figure 2.

During the testing procedure, various measurement instruments were utilized. The SIARGO MF5712 flowmeter (Siargo, Santa Clara, CA, USA) served as the primary tool for measuring flow rates and was cross-referenced with a trusted calibration standard, the TSI 4000 series Mass Flowmeter (TSI Incorporated, Shoreview, MN, USA). To assess oxygen concentration accurately, the digital oxygen measuring instrument CY-12C (CJCMALL, Wuxi, China) was employed and compared with the calibration standard MiniOX I—Model 473030 (MSA, Pittsburgh, PA, USA). The HIT-1890 differential pressure sensor (HIT, Nangang, Harbin, Heilongjiang, China) was used to measure and compare pressure values with the calibration standard TSI 4000 series Mass Flowmeter (TSI Incorporated, Shoreview, MN, USA).

To ensure the integrity and effectiveness of the test setup, two HEPA-type antimicrobial filters, a vent hose, and a 10 cmH_2_O PEEP valve were incorporated. These components were essential in creating a controlled environment and facilitating accurate measurements throughout the testing process.

**Figure 3:**
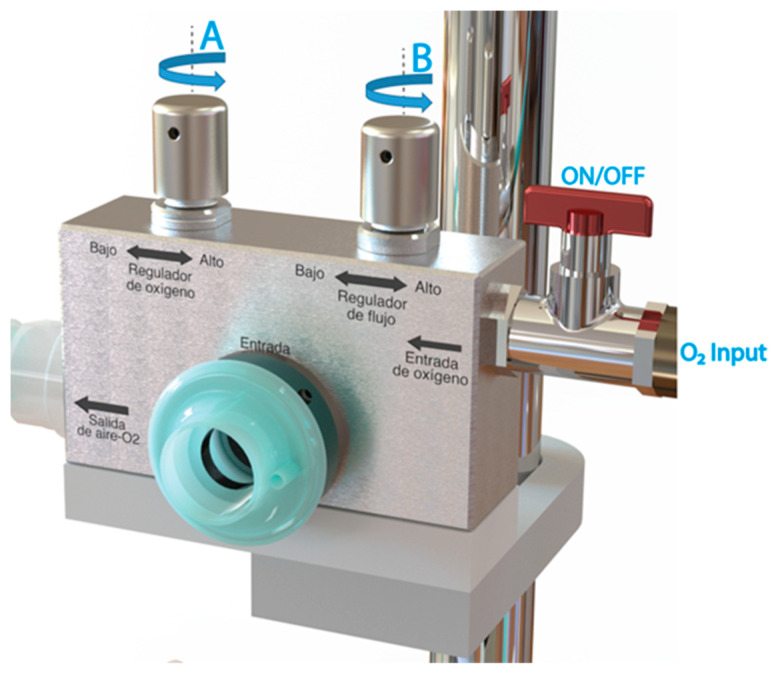
CPAP-AirFlife™ System flow generator valves.

Before initiating the characterization process, it is crucial to ensure that the flow generator valves, namely, valves A and B, are fully closed, as depicted in Figure 3. Additionally, the oxygen supply, typically regulated between 50 and 60 psi, must be prepared from a cylinder or a dedicated line. To commence the procedure, the O_2_ supply valve to the system is opened by activating the ON/OFF switch. Subsequently, the flow regulator valve (valve B) is gradually opened to achieve specific flow levels of 15 L/min, 30 L/min, 45 L/min, and 60 L/min. At each attained flow level, the corresponding minimum (valve A fully closed), 60%, 80%, and maximum (valve A fully open) FiO_2_ values are evaluated. Combining different flow rates with the predetermined medical oxygen concentrations mentioned above. Throughout this process, the flow and oxygen concentration values are meticulously recorded. To ensure the reliability and consistency of the results, the medical oxygen and air control valves (valves A and B) are closed again, allowing for the repetition of the entire process to assess the repeatability of the measurements.

### 2.3. Clinical Trial Design

To ascertain the clinical efficacy of the device, a rigorous randomized non-inferiority and randomized crossover clinical trial was conducted at Servicios Especiales de Salud (S.E.S) Hospital Universitario de Caldas, situated in Manizales, Caldas, Colombia. The testing procedures took place within a specially adapted hospital environment to cater to COVID-19 patients. For comparative analysis of ventilation technologies, the S.E.S Hospital Universitario de Caldas provided the General Electric ventilator model R860 alongside the CPAP-AirFlife™ system.

#### 2.3.1. Outcomes

The main focus of this study was to assess the primary outcome variable, SpO_2_ (peripheral oxygen saturation). Additionally, secondary outcome variables included exhaled CO_2_ levels and the comfort experienced by participants with each CPAP method. These measures were crucial in evaluating the effectiveness and tolerability of the different CPAP techniques employed in this research. 

#### 2.3.2. Eligibility Criteria for Participants

- Inclusion criteria: healthy adult volunteers aged 18 years or older.
- Exclusion criteria: individuals with any form of respiratory pathology or other underlying comorbidities.

#### 2.3.3. Interventions

**Figure 4:**
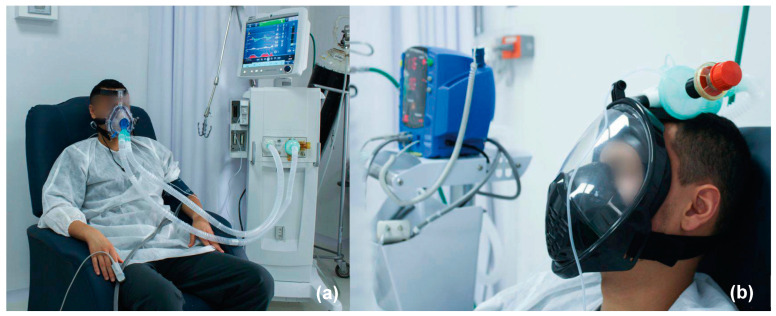
Comparison between conventional (**a**) CPAP and (**b**) CPAP-AirFlife™.

The study included a sample of 19 healthy adult volunteers, who were divided into two groups. Group 1 initiated with conventional CPAP (Figure 4a) followed by CPAP-AirFlife™ (Figure 4b), while Group 2 started with CPAP-AirFlife™ and concluded with conventional CPAP. Each group underwent a 60 min CPAP session according to the randomized order. Subsequently, after a 15 min “wash-out” period the participants engaged in another 60-min session with the CPAP method that differed from the initial one.

#### 2.3.4. Sample Size

The objective of this experimental validation is not to establish the superiority of CPAP-AirFlife™ over a conventional CPAP delivery device (such as the General Electric model R860 ventilator) but rather to demonstrate that it is not inferior or of lower quality than the latter. Based on the null hypothesis Ho, which assumes that CPAP-AirFlife™ is inferior to conventional CPAP $\left(\mu_{A}-\mu_{B}\leq -d_{NI}\right)$, and the alternative hypothesis that CPAP-AirFlife™ is not inferior to conventional CPAP $\left(\mu_{A}-\mu_{B}>-d_{NI}\right)$, we defined the average outcome of CPAP-AirFlife™ therapy and the average outcome of conventional CPAP therapy. To determine the necessary sample size to detect a difference between the means (with a non-inferiority margin), we employed the SampSize application [42]. Our calculations utilized a reliability index of 90%, a significance level of 0.05, a mean difference of 0.05, and a population standard deviation of 0.1. Accordingly, a sample size of 19 healthy adults was determined as sufficient to demonstrate non-inferiority between the two treatment approaches.

#### 2.3.5. Randomization

The randomization process for determining the starting order of each CPAP technology (conventional vs. CPAP-AirFlife™) was conducted using a simple randomization method. This method ensured that each patient had an equal probability of beginning with either option. To maintain the concealment of randomization, sequentially numbered envelopes that were non-transparent were used. An external epidemiologist generated the sequence of randomization. The healthy adult volunteers who participated in the trial were enrolled in the study and kept masked until the assignment of the starting order for each CPAP technology was determined. 

### 2.4. Statistical Methods

For this study, continuous data such as age were reported as means and standard deviations following a normal distribution based on the Shapiro–Wilk test. These data were compared using Student’s *t*-test. The distribution of categorical data was presented and analyzed using Fisher’s exact test. To determine the 95% confidence interval for the difference in mean SpO_2_ at 0, 30, and 60 min after initiating CPAP, Equation (1) was used:

(1) $$(m_{1}-m_{2})\pm 1.96\sqrt{\frac{s_{1}{}^{2}}{n_{1}}+\frac{s_{2}{}^{2}}{n_{2}}}.$$

where *m*_1_ and *m*_2_ represent the observed means in the test period (CPAP-AirFlife™) and active control period (conventional CPAP), respectively, *s*_1_ and *s*_2_ are the corresponding standard deviations, and *n*_1_ and *n*_2_ are the sample sizes for each period. Because there are no clinical studies comparing mean SpO_2_ between patients receiving conventional nasal CPAP and a placebo, the non-inferiority margin (δ) was determined based on clinical criteria. Non-inferiority was considered established if the lower limit of the 95% confidence interval was above the non-inferiority margin (−5%). Furthermore, the 95% confidence interval for the difference in CPAP-associated performance problems was calculated using Equation (2):

(2) $$p_{1}-p_{2}\pm 1.96\sqrt{\frac{p_{1}(1-p_{1})}{n_{1}}+\frac{p_{2}(1-p_{2})}{n_{2}}}.$$

Here, *p*_1_ and *p*_2_ represent the observed proportions of CPAP-associated performance problems in the test (CPAP-AirFlife™) and active control (conventional CPAP) periods, respectively, and y represents the sample size for each period. As there are no clinical studies comparing the rate of CPAP-associated performance problems between patients receiving conventional nasal CPAP and placebo, the non-inferiority margin ($-d_{NI}$) was determined based on clinical criteria. Non-inferiority was considered to be established if the lower limit of the 95% confidence interval was above the non-inferiority margin (−0.8). Data analysis was performed using Stata 16.1 (StataCorp LLC, College Station, TX, USA). 

## 3. Results and Discussion

### 3.1. Experimental Validation CPAP-AirFlife™

Table 1 presents the results of the validation tests conducted on the flow generator of the CPAP-AirFlife™ device, as described in Section 2.2. The validation process involved measuring the flow at four reference points: 15, 30, 45, and 60 L/min, which were the desired flow parameters. The achieved flow values were obtained by adjusting the flow valve and the resulting pressure from the PEEP valve (set at 10 cmH_2_O) was recorded after the air-O_2_ mixture. Overall observations indicate that as the flow rate increases, the PEEP pressure rises; this occurs because the volume of the mixture accumulates and cannot be fully evacuated at the desired outflow rate. The measured pressure exhibits a variability ranging from a minimum of 10.3 cmH_2_O to a maximum of 13.6 cmH_2_O, representing a 25% change in flow. Notably, the average achieved flow closely approximated the desired flow, with a less than 1% variation.

Table 2 shows the results of the measurements conducted to assess the device’s capacity for providing different oxygen (O_2_) concentrations at various flow levels. The oxygen concentrations are expressed as percentages. These measurements aimed to determine the device’s performance in delivering both minimum and maximum oxygen concentrations. Upon analyzing the results, it is evident that as the desired flow rates increase, the maximum and minimum oxygen concentrations decrease.

The measurements revealed interesting findings regarding the variations in minimum and maximum oxygen (O_2_) concentrations at different flow levels. Specifically, a 13% decrease in the minimum O_2_ concentration was observed during the first flow increment from 15 to 30 L/min. However, this measurement remained stable in the subsequent flow variations. On the other hand, the maximum oxygen concentration remained consistent in the first three flow variations (15 to 45 L/min) and showed a decline of approximately 15.5% at the maximum measured flow. These results highlight the significance of considering flow levels when determining oxygen concentration.

**Figure 5:**
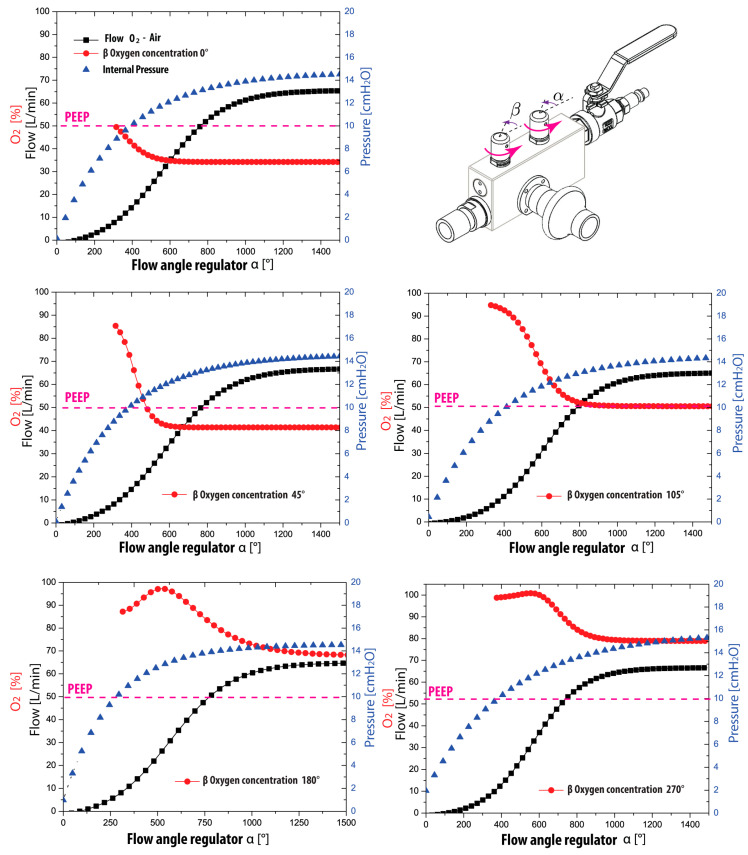
Variation of O_2_-air parameters for different control valve configurations.

A continuous depiction of the measurements conducted to evaluate the flow regulation and oxygen concentration parameters is shown in Figure 5. These results offer valuable insights into the variability of these parameters, which are crucial for establishing functional characteristics and ensuring the quality of the flow generator. These findings hold particular importance when considering the commercialization process of the flow generators. It is essential to emphasize that the validation of the flow generator should be based on assessing three variables: flow, O_2_ concentration, and positive end-expiratory pressure (PEEP). These obtained results play a critical role in guaranteeing the quality of the final product. Additionally, Figure 5 presents valuable information regarding the relationship between flow (represented by the black line) and oxygen concentration (indicated by the red line) and how angular variations influence them in terms of flow opening and O_2_. The graph illustrates that oxygen flow begins to pass at a flow opening angle of 100°. Furthermore, it demonstrates that an O_2_ concentration value is obtained from a flow rate of 10 L/min, highlighting the importance of considering the flow rate to ensure appropriate oxygen delivery.

To achieve the minimum oxygen concentration without adjusting the O_2_ concentration regulating valve, the flow regulating valve must be turned to reach 600°, which corresponds to almost two complete rotations. Notably, the PEEP pressure consistently maintains its behavior across all cases, indicating its independence from the O_2_ concentration variable. However, due to the closed nature of the circuit it is essential to acknowledge that the PEEP value may be exceeded in this scenario. This is because the ventilatory circuit lacks the pressure gradients generated by the patient’s breathing. Under normal circumstances, the patient would be expected to reach the suggested PEEP pressure during exhalation. It is worth mentioning that when the circuit incorporates the mask and the patient receives ventilatory support, the baseline PEEP pressure is achieved during the exhalation process.

The CPAP-AirFlife™ flow regulator possesses a significant advantage in its ability to operate independently of medical air, instead utilizing ambient air to produce air-O_2_ mixtures. By opening the O_2_ regulating valve, it becomes apparent that flow rates below 15 L/min yield oxygen concentrations surpassing 80%. As the flow rate escalates, the air suction intensifies, facilitating precise management of the O_2_ concentration. In practical application, the flow generator exhibits exceptional stability and effortless control, empowering users to establish desired values for flow and oxygen concentration parameters effortlessly.

### 3.2. Clinical Trial in Healthy Patients with the Use of CPAP-Airflife 

**Figure 6:**
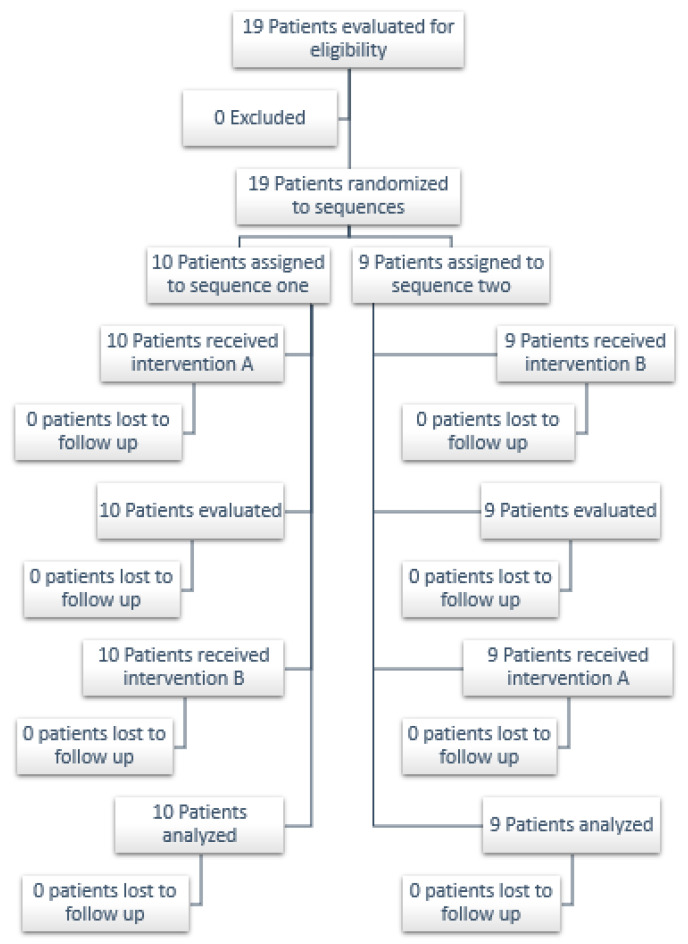
Clinical trial protocol for healthy patients.

This section presents the results of a clinical trial involving 19 healthy patients (see Figure 6) to assess the efficacy of the CPAP-AirFlife™ device compared to a conventional device in maintaining oxygen saturation levels during various treatment durations. While numerous studies have explored the effectiveness of conventional CPAP devices, limited research exists on the efficacy of CPAP devices utilizing oxygen flow generators as the primary ventilation source. Hence, this clinical trial aims to validate the use of CPAP-AirFlife™ in healthy patients through a non-inferiority test, following the approach of previous studies [43]. The objective is to provide a technological solution for future research on its application in patients with respiratory conditions. All trials adhered to the Consort 2010 flowchart for cross-over clinical trials [44]. Table 3 outlines the clinical and demographic characteristics of the sample, revealing a group of 19 relatively young participants with a mean age of 29.5 ± 1.8 years.

The percentage of patients identifying as biological males was 52.6%, against 47.4% for biological women. There was no statistically significant difference in biological sex between the two groups. Regarding smoking status, most participants (73.6%) reported not actively smoking, and there was a slight trend towards a higher percentage of active smokers in Group 1 compared to Group 2. No significant differences were observed between the two groups regarding age, smoking status, former smoking, physical activity, or comorbidities. Furthermore, there were no notable variations in the variables of ex-smokers and physical activity. It is essential to highlight that the reported *p*-values associated with each statistical test accurately assess the significance of the observed differences. Overall, the sample appears similar between the two groups, with no significant disparities in the analyzed demographic and clinical variables. This indicates that these variables are unlikely to impact the observed differences in outcomes, suggesting that the disparities observed between the two groups are more likely attributed to the comparison of CPAP therapies.

The oxygen concentration levels at three different time points after ventilation starts (0, 30, and 60 min) for each group and therapy are shown in Table 4. The study found no significant differences between the two CPAP therapies regarding their impact on oxygen saturation levels in the participants (Figure 4). In the conventional CPAP group, SpO_2_ levels ranged from 93.8% to 99.4%, while in the CPAP-AirFlife™ group they ranged from 94.1% to 99.3%. The confidence intervals indicate that the results are statistically significant.

These results suggest that the CPAP- AirFlife™ device may be an effective alternative to conventional equipment to maintain SpO_2_ levels in patients with respiratory problems. However, further studies are needed to confirm these findings. 

Table 5 presents the comparative analysis of comfort levels between the conventional and AirFlife CPAP therapies in the two participant groups. In Group 1, after 30 min of therapy 60% of participants reported being comfortable with the conventional therapy, while 40% reported the same for the CPAP-AirFlife™ therapy. There was no statistically significant difference in comfort between the two therapies in Group 1. Tolerable discomfort was reported by 40% of participants for the conventional therapy and 50% for the AirFlife therapy. One participant in Group 1 experienced difficulty bearing discomfort with the AirFlife therapy. In Group 2, a higher percentage of participants found the conventional therapy to be comfortable compared to the AirFlife therapy. However, the difference was not statistically significant. Overall, the findings suggest no significant difference in comfort levels between the conventional and AirFlife therapies in either group.

On the other hand, it is essential to highlight that no adverse effects were found after analyzing the data from the 19 cases. This suggests that both CPAP therapies (conventional nasal CPAP and AirFlife CPAP) may be safe and well-tolerated in this patient population. It is important to continue monitoring for potential adverse effects in larger patient populations to validate these findings further. Overall, the lack of adverse effects in this study is promising and supports the use of the AirFlife CPAP therapy in treating respiratory conditions.

## 4. Conclusions

The study findings demonstrate that the low-cost CPAP-AirFlife™ CPAP device incorporating aerosol control headgear is not inferior to conventional equipment across various efficacy measures. These measures include hemoglobin oxygen saturation levels, exhaled PCO_2_ levels, vital signs, and individual tolerance. These results highlight the potential of the CPAP-AirFlife™ device as a viable and effective alternative for treating patients with respiratory distress.

One significant advantage of the CPAP-AirFlife™ device is its accessibility, particularly in resource-limited areas and lower-level hospitals where the cost and availability of treatments present significant challenges. The device’s affordability and local availability can significantly improve access to effective respiratory treatments, particularly in rural regions. This has profound implications for enhancing the quality of life and overall health of individuals with respiratory conditions requiring specialized care.

Moreover, the portability and ease of use of the CPAP-AirFlife™ device make it a suitable option for patients needing CPAP therapy during transportation to specialized medical facilities. Its independence from external sources of medical air or electricity, along with its ergonomic and user-friendly design, allow for straightforward operation without requiring specialized personnel. Consequently, the CPAP-AirFlife™ device emerges as an efficient and convenient alternative for providing respiratory support in emergencies and during patient transfers to other medical centers.

In summary, this study underscores the CPAP-AirFlife™ device as a viable and effective solution for addressing respiratory problems, particularly in resource-constrained areas. Its affordability, accessibility, and portability make it a valuable tool in improving respiratory care and overcoming the challenges faced by patients in underserved regions. By bringing effective respiratory treatments within reach, the CPAP-AirFlife™ device has the potential to improve the well-being of individuals in need significantly.

## Figures and Tables

**Table 1:** Variations in the flow and pressure parameters.

Desired Flow (L/min)	Measured Flow (L/min)	σ (L/min)	Measured Pressure (cmH_2_O)	σ (L/min)	Reference Pressure (cmH_2_O)
15	15.6	0.3	10.3	0.3	10
30	30.6	0.3	11.3	0.2	10
45	45.8	0.2	12.5	0.3	10
60	60.3	0.4	13.6	0.2	10

**Table 2:** Variations in Oxygen (O_2_) concentration.

Oxygen Concentration				
Measured Flow (L/min)	Minimum O_2_ $\bar{y}$ (%)	σ (%)	Maximum O_2_ (%)	σ (%)
15.5	41.5	0.8	99.7	0.2
30.6	36.2	0.7	99.6	0.3
45.7	34.2	0.4	97.3	0.5
60.8	34.0	0.3	82.2	0.6

**Table 3:** Clinical and sociodemographic characteristics.

Variable	Group 1	Group 2	*p*
Gender: Male Female	7 (70%): 3 (30%)	3 (33.3%): 6 (66.7%)	0.17 *
Age	28.7 ± 1.7	30.3 ± 1.9	0.54 **
Current smoker:			0.30 *
Yes	4 (40%)	1 (11.1%)	
No	6 (60%)	8 (88.9%)	
Former smoker:			0.46 *
Yes	2 (25%)	0 (0%)	
No	6 (75%)	8 (100%)	
Physical activity:			0.46 *
Yes	2 (25%)	0 (0%)	
No	6 (75%)	8 (100%)	
Comorbidity:			0.46 *
Yes	2 (25%)	0 (0%)	
None	6 (75%)	8 (100%)	

* Fisher’s exact test ** Student’s *t*-test.

**Table 4:** SpO_2_ by type of CPAP and study group.

			Group	
Tipe de CPAP	Time [min]	IC	1	2
Conventional	0	X	95.5	93.8
		95%	94.6–96.5	92.2–95.3
	30	X	99.3	98.9
		95%	98.6–99.9	98.3–99.4
	60	X	99.4	99.3
		95%	98.6–100.2	98.8–99.7
CPAP-AirFlife	0	X	95.2	94.1
		95%	94.0–96.3	92.3–95.8
	30	X	99	98.4
		95%	98.2–99.7	96.8–99.9
	60	X	99.3	98.8
		95%	98.5–100.1	97.3–100.3

**Table 5:** Qualitative results for comfort.

Group	Comfort after 30 min	Conventional		AirFlife		p *
		n	%	n	%	
1	Comfortable	6	60.0	4	40.0	0.58
	Tolerable discomfort	4	40.0	5	50.0	
	Difficult to bear discomfort	0	0	1	10.0	
	Unbearable discomfort	0	0	0	0	
2	Comfortable	7	77.8	2	22.2	0.33
	Tolerable discomfort	2	22.2	7	77.8	
	Difficult to bear discomfort	0	0	0	0	
	Unbearable discomfort	0	0	0	0	

* Fisher’s exact test.

## Data Availability

The data collected in this research are available when be required.
